# A Novel Functional TagSNP Rs7560488 in the DNMT3A1 Promoter Is Associated with Susceptibility to Gastric Cancer by Modulating Promoter Activity

**DOI:** 10.1371/journal.pone.0092911

**Published:** 2014-03-25

**Authors:** Huazhang Wu, Kun Zhang, Pihai Gong, Fengchang Qiao, Ling Wang, He Cui, Xinyuan Sui, Jifan Gao, Hong Fan

**Affiliations:** 1 The Key Laboratory of Developmental Genes and Human Diseases, Ministry of Education, Departments of Medical Genetics and Developmental Biology, Medical School of Southeast University, Nanjing, China; 2 The Third Affiliated Hospital of Harbin Medical University (Harbin Medical University Cancer Hospital), Harbin, China; The University of Hong Kong, China

## Abstract

DNA-methyltransferase (DNMT)-3A which contains *DNMT3A1* and *DNMT3A2* isoforms have been suggested to play a crucial role in carcinogenesis and showed aberrant expression in most cancers. Accumulated evidences also indicated that single nucleotide polymorphisms (SNP) in DNMT genes were associated with susceptibility to different tumors. We hypothesized that genetic variants in *DNMT3A1* promoter region are associated with gastric cancer risk. We selected the tagSNPs from the HapMap database for the Chinese and genotyped in a case-control study to evaluate the association with gastric cancer (GC) in a Chinese population. We identified that the functional tagSNP rs7560488 T>C associated with a significantly increased risk of GC. *In vitro* functional analysis by luciferase reporter assay and EMSA indicated that the tagSNP rs7560488 T>C substantially altered transcriptional activity of *DNMT3A1* gene via influencing the binding of some transcriptional factors, although a definite transcriptional factor remains to be established. Compared with TT homozygotes, subjects who were TC heterozygotes and CC homozygotes exhibited a reduced expression of *DNMT3A1*. Furthermore, stratified analysis showed that individuals who harbor TC or CC genotypes less than 60 years old were more susceptible to GC. Our results suggest that the genetic variations in the *DNMT3A1* promoter contribute to the susceptibility to GC and also provide an insight that tagSNP rs7560488 T>C may be a promising biomarker for predicting GC genetic susceptibility and a valuable information in GC pathogenesis.

## Introduction

Gastric cancer is one of the most common malignant tumors in China, especially in Jiangsu province with a high incidence and mortality rate [1], [2]. It can spread throughout the stomach and to other organs, including the esophagus, lungs, lymph nodes or liver. Therefore, gastric cancer is the second leading cause of cancer-related death in the world [3]. In consideration of the therapeutic efficiency, surgical resection can be a primary curative treatment for earlier stage of GC patients [4]. Unfortunately, most gastric cancer patients are detected in advanced stage, during which period the tumor are unresectable anymore. Furthermore, relapse after surgery is another terrible event for a poor 5-year survival rate. Considering the patients with advanced or recurrent gastric cancer, it is no doubt that discovery of biomarkers and their application accompanied with traditional diagnosis might be a valuable indication and an extensive help to formulate the prevention and treatment strategy. However, so far, few measurable biomarkers for predicting GC recurrence have been identified.

Tumorigenesis is known to be a multistep process, which is the result of not only genetic alterations but also epigenetic changes [5]. DNA methylation is a major form of epigenetic modification and plays an essential role in development, differentiation, genomic stability, X-inactivation, and imprinting by specific regulation of gene expression. The most commonly studied epigenetic phenomenon is DNA methylation, an essential regulator of transcription and chromatin structure. Aberrant DNA methylation patterns in a genetically susceptible background may be associated with increased risk of a series of human disorders [6],[7], including GC [8]. *DNMT3A* which contains *DNMT3A1* and *DNMT3A2* are two *de novo* DNA methyltransferases plays a crucial role in embryonic development and aberrant DNA methylation in carcinogenesis. Some polymorphisms of the *DNMT3A* gene may regulate gene expression, influence its enzymatic activity and may contribute to susceptibility to cancer. Accumulated evidences in molecular genetics indicate that SNP in *DNMT* genes are associated with susceptibility to cancer [9], [10]. Recent progresses in genome-wide association study (GWAS) also have been identified new susceptibility SNPs for GC, which is helpful to understand the underlying mechanism of genetic variations in the development of GC [11]–[14]. Our previous study found a functional SNP rs1550117 in *DNMT3A* promoter that can increase its transcriptional activity and contribute to the genetic susceptibility to gastric cancer in a Chinese population [15], [16].

GWAS has yielded numerous SNPs associated with many cancers. In some cases, dozens of SNPs, called tagSNPs which represent SNPs in a region of the genome with high linkage disequilibrium can identify genetic variation without genotyping every SNPs in a chromosomal region, so tagSNPs are useful in whole-genome SNP association studies, such as prostate, breast, ovarian, colorectal and brain cancers [17]–[19]. In the present study, we selected a tagSNP rs7560488 from the HapMap database for Chinese subjects to evaluate the associations between the genetic variants in the *DNMT3A1* promoter and gastric cancer risk in a Chinese population. We identified a risk-associated rs7560488 T>C polymorphism in the *DNMT3A1* promoter, and our further work suggested that this variant could alter the promoter activity and destroy the binding ability of transcriptional factors.

## Materials and Methods

### Study Subjects

A total of 405 patients with histologically confirmed gastric cancer and 408 cancer-free controls were recruited in this case-control study, and the characteristics of the cases and controls are detailed in Table 1. Cases and controls were matched by age, sex and were selected from the First Affiliated Hospital of Nanjing Medical University. All of the samples were obtained with written consent and analyzed anonymously. This study was performed with the approval of the Medical Ethical Committee of Medical School of Southeast University.

**Table 1:** Characteristics of the study population.

Variables	GC Cases (n = 405)	Controls (n = 408)	*P* value^a^
Age (years)			
≤60	180 (44.4%)	198 (48.5%)	0.243
>60	225 (55.6%)	210 (51.5%)	
Gender			
Male	289 (71.4%)	279 (68.4%)	0.355
Female	116 (28.6%)	129 (31.6%)	

a Two-sided χ2 test for genotype distribution.

### TagSNP Selection and the TF Binding Site Prediction

The principal hypothesis underlying this experiment was that there are one or more SNPs in the *DNMT3A1* promoter regions that are associated with the risk of gastric cancer. Depending on the linkage disequilibrium (LD) structure at a particular locus, tagSNPs may be surrogates for many thousands of other SNPs. We postulate that such tagSNPs are also likely to tag any hitherto identified SNPs in the *DNMT3A1* promoter. Thus, we selected the SNPs in the *DNMT3A1* promoter region with a minor allele frequency (MAF) of >5% from both the HapMap and dbSNPs databases. To implement potentially functional tagSNP selection, we use data from the International HapMap and the freely web-based tagSNP selection tools to select tagSNPs, and use the TF-search algorithm (http://mbs.cbrc.jp/research/db/TFSEARCH.html) to predict rs7560488 transcription factor (TF) binding site.

### DNA Extraction and HRM Genotyping

To study the *DNMT3A1* promoter tagSNP rs7560488, genomic DNA was isolated from 1 ml of peripheral blood from patients and healthy individuals and was extracted from white blood cells within a week after sample collection by proteinase K digestion as previously described [20]. TagSNP rs7560488 was genotyped using the dsDNA dye LC Green in combination with High Resolution Melting (HRM) analysis. In detail, the PCR primers were designed by the LightScanner primer design software (Idaho Technology) (forward primer: 5′-AGGCAGACACAAATGCATAAAT-3′; Reverse primer: 5′-GTCATAAGTACAACCACCACCG-3′) which product a single 208 bp fragment. Each PCR reaction was initially performed in a final reaction volume of 10 μL, using 25 ng of genomic DNA, 0.2 pmol of each primer, 0.8 μL 2.5 mM dNTPs, 1 μL 25 mM MgCl_2_, 1 μL 10×Taq buffer with (NH4)_2_SO_4_, 0.4 U Taq DNA Polymerase (Fermentas), 1 μl 1X LC Green PLUS (Idaho Technology) and 0.4 μL dimethyl sulfoxide (DMSO). The reaction mixture was incubated at 95°C for 5 min and then subjected to 40 cycles of 95°C for 30 sec, 57°C for 30 sec, and 72°C for 30 sec, followed by 72°C for 7 min using a PTC-200 thermal cycler (Bio-Rad). The PCR reactions were transferred to the 96-well plates (Bio-Rad) and analyzed on the Light Scanner (Idaho Technology). Fluorescence data were collected over a temperature range of 70–97°C, and melting curve analysis was performed according to the manufacturer’s software. HRM could directly discriminate the heterozygote (TC) and homozygote (CC or TT) genotypes of tagSNP rs7560488 T>C through melt scanning. After mixing homozygous DNA with an equal amount of known PCR products (e.g., CC), it further distinguished between the CC and TT genotypes. For further confirmation, 5% of samples from each group detected by HRM were randomly selected and subjected to DNA sequencing to ensure reliability and reproducibility.

### Construction of Luciferase Reporter Plasmid

To construct the DNMT3A1 tagSNP rs7560488 reporter plasmid, we amplified the 948 bp fragment from 25422345 to 25422345 of *DNMT3A1* promoter region, which contains the T and C allele of SNP by PCR from genomic DNA. The primers used for the PCR amplifications were: (Forward: 5′-TACGCTAGCATACCAAGTCCCCATTCCCC-3′, Reverse: 5′-GTATAAGCTTTCGGCTTCTACACCCCTCAC-3′). The PCR products were subcloned into the NheI and HindIII restriction sites of the pGL3-Basic vector (Promega, Madison, WI). We verified all recombinant clones by DNA sequencing.

### Transient Transfection and Dual Luciferase Reporter Assay

Human gastric cancer AGS and BGC-823 cells (ATCC) were grown in RPMI-1640 medium supplemented with 10% Fetal Bovine Serum (FBS) and 1% penicillin/streptomycin solution (10 000 U/mL and 10 mg/mL, respectively). AGS and BGC-823 cells (1×10^5^) were seeded in 24-well culture plates. After 24 hours of culture, AGS, BGC-823 cells were transfected by Lipofectamine 2000 (Invitrogen, Carlsbad, CA, USA) with 0.8 mg of each constructed vector, either with T allele or C allele. Simultaneously, 10 ng pRL-TK plasmids (Promega) per well was also transfected as an internal control for correcting transfection efficiency. Before it, cells were seeded on 24-well plates over night to ensure 90%–95% confluence at the time of transfection. Twenty-four hours after transfection, luciferase activity was measured by the Dual-Luciferase Reporter Assay System (Promega, Madison, WI, USA) and expressed as the ratio of Firefly luciferase to Renilla luciferase activities. All cells were done in triplicate with the same conditions. Three independent transfection experiments were performed, and each luciferase assay was carried out in triplicate.

### Electrophoretic Mobility Shift Assay (EMSA)

The 5′-biotinylated oligos 25 bp in length were obtained from Beijing Genomics Institute (BGI). Oligo sequences were rs7560488 [T] Forward: 5′-TAGTCAGACTCATAGAGACAGAAG-3′, rs7560488 [T] Reverse: 5′-CTTCTGTCTCTATGAGTCTGACTA-3′. rs7560488[C] Forward: 5′-TAGTCAGACTCACAGAGACAGAAG-3′, rs7560488[C] Reverse: 5′-CTTCTGTCTCTGTGAGTCTGACTA-3′. For annealing, concentrated complementary oligonucleotides were mixed at a 1∶1 molar ratio and incubated at 95°C for 5 min and then gradually reduced over hours until the oligonucleotides reached room temperature. Annealed oligos were diluted to a final concentration of 10 fmol. Nuclear proteins were extracted from BGC-823 cells using the NE-PERTM Nuclear and Cytoplasmic Extraction Reagents (Pierce, Rock-ford, IL, USA) according to the manufacturer’s instructions. The LightShift Chemiluminescent EMSA kit (Pierce/Thermo Fisher Scientific) was used according to the manufacturer’s instructions. Briefly, binding reactions were performed as follows: nuclear extracts (8 μg protein) and the 1× binding buffer with 2.5% glycerol, 5 mM MgCl2, 50 ng/μl poly (dI-dC), 0.05% NP-40, and 60 fmol biotin-labeled rs7560488 T/rs7560488 C probes were incubated on ice for 30 min in a volume of 20 μl. For competition studies, nuclear extracts were incubated with unlabeled oligonucleotide for 30 min before the addition of labeled oligonucleotide. For a supershift, AP-1 antibody was added (BOSTER, China). Complexes were separated by electrophoresis on native 6% PAGE in 0.5× TBE buffer at 110 V. Gels were transferred to Biodyne B pre-cut modified nylon membranes (pierce/Thermo Fisher Scientific) using a Trans-Blot SD semi-dry transfer cell (Bio-Rad Laboratories). Membranes were cross-linked (UVC-508 UV Cross-linker, Ultra LUM) and the signal was detected with a chemiluminescent detection system (Pierce/Thermo Fisher Scientific) according to the manufacturer’s instructions.

### Detection of DNMT3A1 Transcripts by Quantitative RT-PCR (Q-PCR)

To further detect the correlation between the DNMT3A1 mRNA levels and rs7560488 polymorphism, the 44 gastric cancer tissues with different genotypes were subjected to extraction of the total RNA using Trizol Reagent (Invitrogen, Inc.). The DNMT3A1 mRNA level was measured by quantitative real-time PCR after reverse transcription on a Prism 7900 Real-Time PCR machine (Applied Biosystems, Foster City, CA). β-actin was used as an internal quantitative control for each sample. The primers used for DNMT3A1 amplification were F: 5′-GAACAGAAGGAGACCAACATCGAA-3′ and R: 5′-GCGCTTGCTGATGTAGTAGGG-3′; the primers for β-actin were F: 5′-GACCTCTATGCCAACACAGT-3′ and R: 5′-AGTACTTGCGCTCAGGAGGA-3′. Relative quantification of DNMT3A1 mRNA was calculated by using the 2-ΔΔCT method, and each assay was done in triplicate.

### Statistical Analyses

All data were analyzed with *SPSS* version 13.0 (SPSS Inc., Chicago, IL, USA). Patients and controls were compared using Student’s *t*-test for continuous variables and chi-square (χ2) test for categorical variables. Allele and genotype frequencies between control and GC subjects were obtained using the chi-square test,and the standard goodness-of-fit test was used to test the Hardy-Weinberg equilibrium. A *P* value of less than 0.05 was considered statistically significant.

## Results

### Characteristics of Study Subjects

The frequency distributions of the cases and controls are presented in Table 1, there was no significant difference in the frequency distributions between the cases and controls (P = 0.243 for age and P = 0.355 for sex). The average of patients and controls was 59.8 years (range 20∼93 years) and 60.6 years (range 25∼90 years), respectively. No significant difference was found in average age and gender, suggesting that matching based on these two variables was adequate.

### Candidate tagSNP Selection and Genotyping

**Figure 1:**
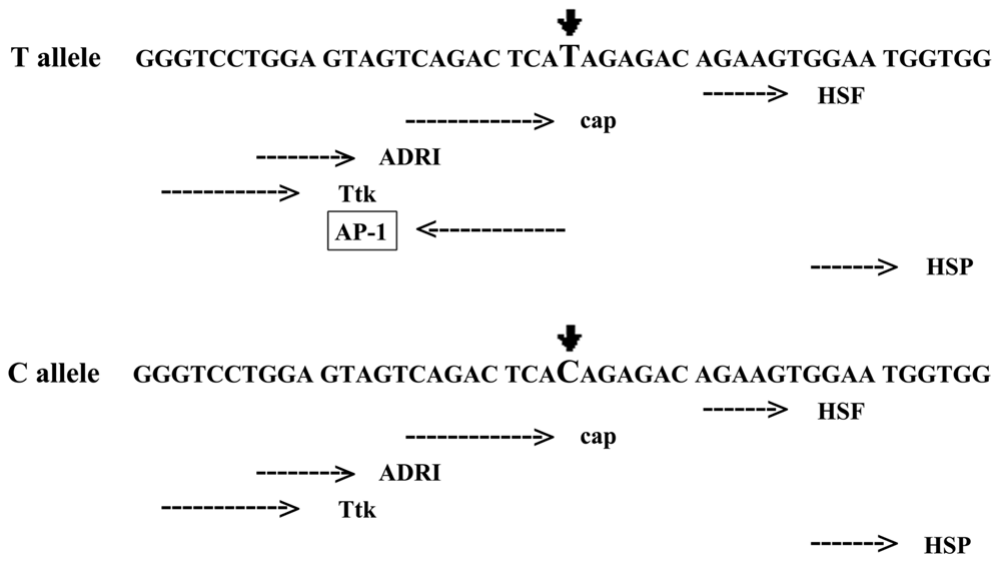
Transcription factor AP-1 was computationally predicted as the possible target transcription factor at the rs7560488 T>C position.

**Figure 2:**
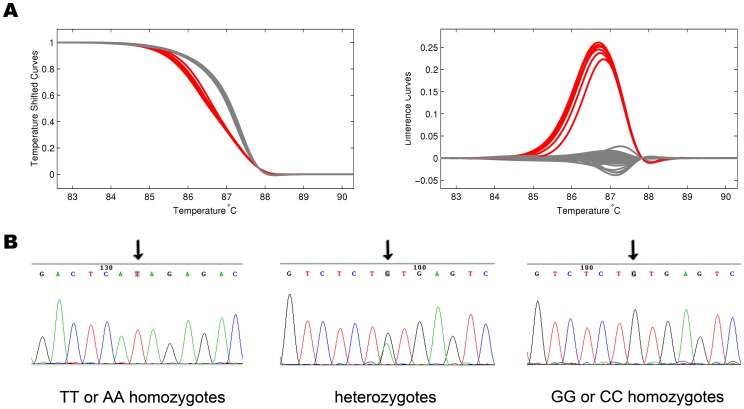
Demonstration of genotyping and sequence. (A) HRM directly discriminated the heterozygotes (TC) and homozygotes (TT or CC), homozygous PCR products (TT or CC) were measured by LightScanner after being mixed with an equal amount of a known product (TT), which distinguished the wild homozygous samples (TT) from the variant ones (CC), as the mutational homozygotes (CC) were converted into heterozygotes (TC). (B) Random samples from rs7560488 T>C testing were sequenced for confirmation, The black arrow indicates the nucleotide polymorphism at the rs7560488 loci.

Among the candidate SNPs in *DNMT3A1*, we focused on the tagSNPs in the promoter of *DNMT3A1* and predicted their potential function on binding transcription factors, which affect the qualitative and quantitative expression of the *DNMT3A1*. We applied a LD-based tagSNP selection algorithm (r^2^≥0.80, MAF≥5%), which identified two tagSNPs representing common genetic variation in CHB population, including the candidate tagSNPsrs7560488 and rs1550117 which is a functional polymorphism that modifies the susceptibility in gastric cancer we confirmed before [15], [16]. TFSEARCH algorithm predicted that rs7560488 T creates a binding site for AP-1 (Figure 1). The samples for genotyping by HRM and sequencing by ABI 3730 automated sequencer respectively (Figure 2).

### TagSNP rs7560488 Variant T>C in DNMT3A1 Promoter Significantly Increases the Risk of GC

The genotype distributions and allele frequencies of rs7560488 are presented in Table 2. The genotype frequencies in the controls were in agreement with the Hardy-Weinberg model (P = 0.274). As shown in Table 2, the genotype frequencies of rs7560488 were 68.9%, 27.4%, and 3.7% for the TT, TC, and CC genotypes among the cases, and 79.9%, 18.4%, and 1.7% among the controls, respectively, the difference between the cases and controls was statistically significant (P<0.05). In addition, the T allele frequency was significantly lower among cases than controls (82.6% versus 89.2%, P = 0.000). In addition, the combined TC/CC genotype frequency was higher among cases than controls (31.1% versus 20.1%, P = 0.002). When taking TT genotype and T allele as reference, we found that the variant genotypes (TC and CC) were associated with an increased risk of GC (OR = 1.653, 95% CI = 1.194–2.287; P = 0.002). Similarly, we also observed that the C allele frequencies was statistically significantly higher than controls (OR = 1.744, 95% CI = 1.310–2.321; P = 0.000). Taken together, these data suggested that the TC and CC genotypes were associated with the genetic susceptibility to GC; the *DNMT3A*1 rs7560488 T allele may be a putative protective allele. There were no significant different frequencies of rs7560488 in GC at age range >60 years versus ≤60 years (P = 0.756), and male versus female (P = 0.459) (Table 3).

**Table 2:** Distribution of genotypes and allele of rs7560488 polymorphism between gastric cases and healthy controls and association with gastric cancer risk.

Genotype/allele	Gastric cancer		Control subjects		OR (95% CI)	*P* value^a^
	n = 405		n = 408			
	No.	%	No.	%		
TT	279	68.9	326	79.9	1	
TC	111	27.4	75	18.4	1.729(1.239–2.414)	0.01
CC	15	3.7	7	1.7	2.504(1.007–6.228)	0.042
TC+CC	126	31.1	82	20.1	1.653(1.194–2.287)	0.002
T	669	82.6	728	89.2	1.744(1.310–2.321)	0.000
C	141	17.4	88	10.8		

a Two-sided χ2 test for genotype distribution.

**Table 3:** DNMT3A rs7560488 T>C genotypes and allele frequencies in GC cases.

Groups	Genotype			Allele		
	TT (%)	TC (%)	CC (%)	T	C	*P* value^a^
Total	279 (68.9)	111 (27.4)	15(3.7)	669 (82.6)	141 (17.4)	
Age						
>60	154 (68.4)	62 (27.6)	9 (4.0)	370 (82.2)	80 (17.8)	0.756
≤60	125 (69.5)	49 (27.2)	6 (3.3)	299 (83.1)	61 (16.9)	
Gender						
Male	203 (70.2)	75 (26.0)	11 (3.8)	481 (83.2)	97 (16.8)	0.459
Female	76 (65.5)	36 (31.0)	4 (3.5)	188 (81.0)	44 (19.0)	

a Two-sided χ2 test for genotype distribution.

### Individuals Less than 60 Years Old were more Susceptible to Gastric Cancer with tagSNP rs7560488 Variant T>C

Age and sex were important factors in tumor carcinogenesis including gastric cancer. When the analyses were stratified by the age and gender of the patients, we found that significant association was observed, individuals carrying TC/CC genotypes were associated with the genetic susceptibility to GC both in male and female group. Therefore, rs7560488 C allele was a significantly increased risk factor compared with T allele (Table 4). Further stratification evaluated the association of rs7560488 T>C with gastric cancer in different ages. TC/CC genotypes were associated with the genetic susceptibility to GC at the age range ≤60 years (OR = 1.794, 95% CI = 1.118–2.877; P = 0.015) other than older than 60, similarly, we also observed that the C allele frequencies was statistically significantly higher than controls (OR = 1.720, 95% CI = 1.127–2.622; P = 0.011). These results suggested that the TC and CC genotypes were associated with the genetic susceptibility to GC, particularly in individuals no more than 60 years (Table 4).

**Table 4:** Stratification analysis of the genotype and allele distribution of rs7560488 and associated odds ratio (OR) in relation to age and gender in GC cases.

Genotype/allele	GC Cases (%)	Controls (%)	OR (95% CI)	*P* value^a^
**Male**				
TT	203 (70.2)	221 (79.2)	1	
TC	75 (26.0)	51 (18.3)	1.601(1.069–2.397)	0.022
CC	11 (3.8)	7(2.5)	1.711(0.651–4.498)	0.271
TC+CC	86 (29.8)	58 (20.8)	1.614(1.100–2.369)	0.014
T	481 (83.2)	493 (88.4)		
C	97 (16.8)	65 (11.6)	1.530(1.090–2.145)	0.013
**Female**				
TT	76 (65.5)	105 (81.4)	1	
TC	36 (31.0)	24 (18.6)	2.072(1.143–3.757)	0.015
CC	4 (3.5)	0 (18.6)	2.382(2.007–2.826)	0.033
TC+CC	40 (34.5)	24 (18.6)	2.303(1.282–4.137)	0.005
T	188 (81.0)	234 (90.7)	2.282(1.339–3.889)	0.002
C	44 (19.0)	24 (9.3)		
**>60** **yrs**				
TT	154 (68.4)	164 (78.1)	1	
TC	62 (27.6)	42 (20.0)	1.572(1.003–2.464)	0.048
CC	9 (4.0)	4 (1.9)	2.396(0.723–7.941)	0.141
TC+CC	71 (31.6)	52 (21.9)	1.454(0.955–2.213)	0.080
T	370 (82.2)	364 (88.1)	1.405(0.970–2.036)	0.071
C	80 (17.8)	56 (11.9)		
**≤60 yrs**				
TT	125 (69.5)	159 (80.3)	1	
TC	49 (27.2)	36 (18.2)	1.731(1.061–2.826)	0.027
CC	6 (3.3)	3 (1.5)	2.544(0.624–10.374)	0.178
TC+CC	55 (30.5)	39 (19.7)	1.794(1.118–2.877)	0.015
T	299 (83.1)	354 (89.4)	1.720(1.127–2.622)	0.011
C	61 (16.9)	42 (10.6)		

a Two-sided χ2 test for genotype distribution.

### The rs7560488 T>C Variant Affects DNMT3A1 Transcriptional Activity

**Figure 3:**
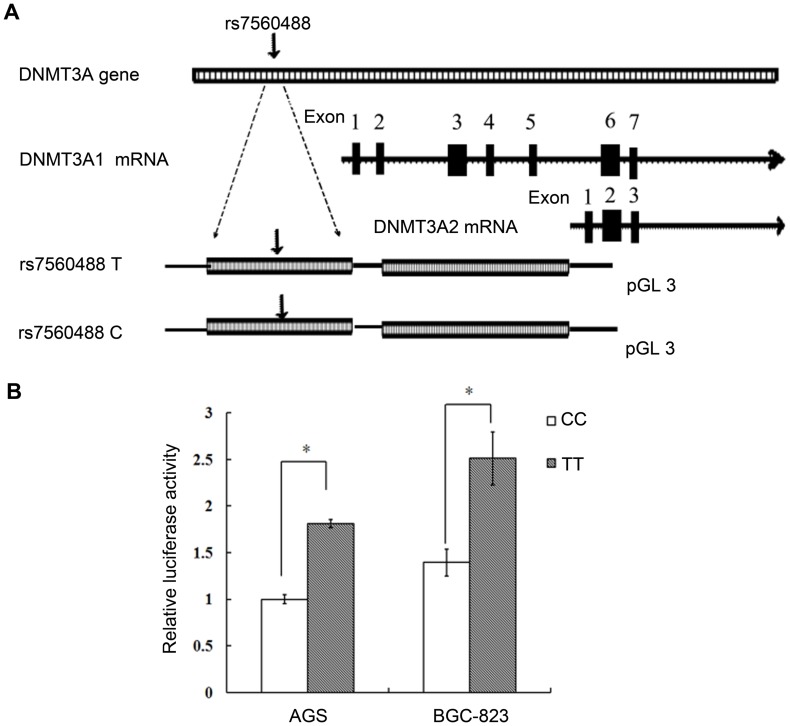
Effect of the rs7560488 T>C polymorphism on DNMT3A1 promoter transcription activity. (A) Schematic representation of reporter plasmids containing the rs7560488 T or rs7560488 C allele, which was inserted upstream of the luciferase reporter gene in the pGL3 basic plasmid. (B) The two constructs were transiently transfected into the AGS and BGC-823 cells respectively. The luciferase activity of each construct was normalized against the internal control of Renilla luciferase. Columns mean from three independent experiments; bars, SD. *, P<0.01 compared with the construct counterpart.

To evaluate the biological functional effect of rs7560488 polymorphism on DNMT3A1 transcription, we constructed luciferase reporter vectors (pGL3), spanning the 4389823 to 4390770 base from *DNMT3A1* promoter, with either wild type (T allele) or mutant type (C allele) and transfected them into BGC-823, AGS cells (Figure 3A). As shown in Fig. 3B, we found that the transcription activity of T allele was higher than C allele with an approximately 2-fold in above two cell lines, suggesting that rs7560488 T allele worked as a defender for gastric cancer by increasing the transcription of *DNMT3A1*.

### The rs7560488 T>C Variant Attenuates Transcription Factor Affinity

**Figure 4:**
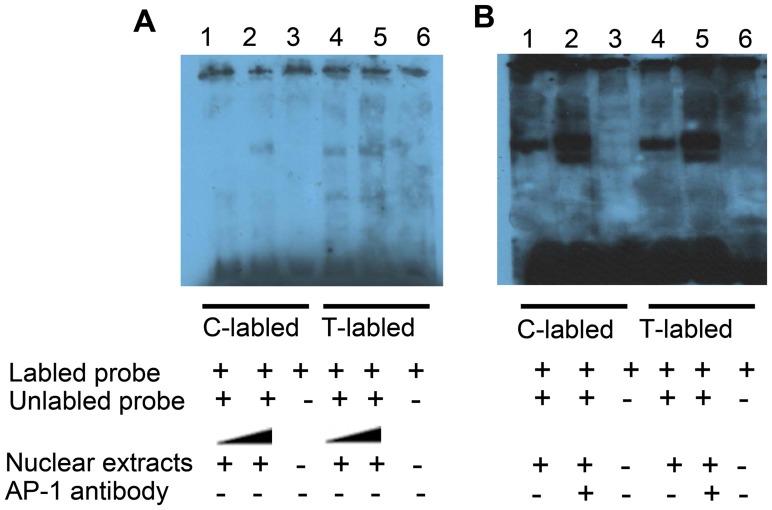
Analysis of transcription factor binding sites in the DNMT3A1 promoter region containing the rs7560488 T>C polymorphism. (A) Nuclear proteins binding activity of different alleles of DNMT3A1 rs7560488 polymorphism. biotinylated probes (60 fmol) were incubated with nuclear extracts from BGC-823 cells. In competition experiments, 50-fold molar excess of unlabeled T or C probes were utilized to demonstrate the specificity of each binding reaction. (B) The super-shift assay conducted using 20 ng anti-AP-1 antibody (lane 2, 5).

In view of tagSNP rs7560488 is located in the *DNMT3A1* promoter region; we hypothesized that it might alter binding of transcription factor (TF). Indeed, using the TF-search algorithm (www.cbrc.jp/research/db/TFSEARCH.html), we predicted that rs7560488 T creates a TF binding site for AP-1. To determine whether this polymorphism has an effect on binding ability of the transcription factor, we conducted the electrophoretic mobility shift assay (EMSA) to analyze the binding of oligo probes containing either T or C allele to nuclear proteins extracted from the AGS cell. As shown in Fig. 4A, a specific shifted DNA/nuclear protein complex band was generated by both C and T allele probes (Fig. 4 A lanes 2, 5). However, T allele still have not been fully competitively inhibited (Fig. 4A lane 4), although the shifted band was abolished by 50-fold unlabeled C probes (Fig. 4A lane 1), suggesting that the binding activity of the sequence containing rs7560488 T allele was stronger compared with C allele and the transcription factor might preferentially bind to the T allele rather than C allele. Moreover, super-EMSA using AP-1 antibody not caused a supershift of the biotin-labeled probe/nuclear protein (Fig. 4 B lane 2, 5) indicating that the AP-1 may not the transcription factor that binds to the promoter region containing the T or C allele. These results indicated that rs7560488 C allele could decrease the nuclear protein binding activity although the impact is not affected by the transcription factor AP-1.

### Association between DNMT3A1rs7560488 Polymorphism and the Expression Levels of *DNMT3A1* mRNA

**Figure 5:**
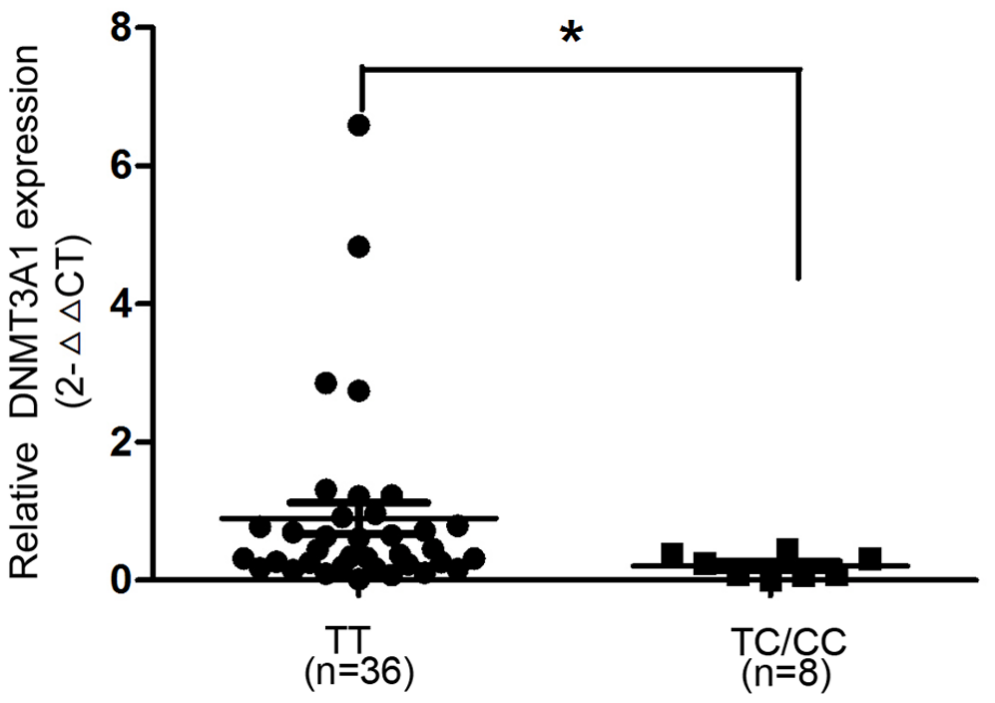
Association between rs7560488 polymorphism in DNMT3A1 promoter region and DNMT3A1 mRNA levels in gastric cancer cases (n = 44). TT versus TC/CC genotypes.

Forty-four gastric cancer tissues with different genotypes of DNMT3A1 rs7560488 were available in our present study. Because of the low frequency of CC genotype, we added it into the samples with TC genotype for analysis. As shown in Fig. 5, the expression levels of DNMT3A1 mRNA was lower in individuals with TC or CC genotype than in those with TT genotype (P<0.05).

## Discussion

Genome-wide hypomethylation and promoter hypermethylation are hallmarks of a great variety of cancers contributing to tumorigenesis and DNA methylation plays key roles in regulating gene expression and maintaining genomic stability [21], [22]. DNA methylation is performed by DNA methyltransferases (DNMTs) *DNMT1*, *DNMT3B* and *DNMT3A* [23], [24]. The *de novo* methyltransferases *DNMT3A* are highly expressed during early embryonic development and down-regulated in most differentiated somatic cells [25]. The role of *DNMT3A* in human cancer was highlighted by reports of *DNMT3A* mutations in approximately 20% of patients with acute myeloid leukemia [26], [27]. The occurrence of these mutations correlated with reduced enzymatic activity and genomic regions with decreased methylation. *DNMT3A* mutations were also identified in 8% of patients with myelodysplastic syndrome [28]. *DNMT3A* also plays a critical role in the epigenetic silencing of hematopoietic stem cell (HSC) regulatory genes and enabling efficient differentiation [29].

The *DNMT3A* genomic locus produces two transcripts giving rise to two proteins, the longer *DNMT3A1* and the shorter *DNMT3A2*, which differ in that a 219-amino-acid amino (N)-terminal tail is present only in *DNMT3A1* [30], [31]. The N-terminal domain of *DNMT3A1* is called a “regulatory” domain because it does not possess enzymatic DNA methyltransferase activity. This domain does not share significant homology with any other known protein. *DNMT3A1* is concentrated in heterochromatin, which is considered to be transcriptionally silent, and functions primarily as a transcriptional repression [30]. But other research showed that *DNMT3A1* was efficiently recruited to the silenced Oct3/4 and activated vitronectin (Vtn) gene promoters via its unique N-terminal domain [32].

It has been reported that genetic variations in the *DNMT3A* gene contribute to carcinogenesis especially associated with GC [15], [33]–[36]. Then, further exploration of the relationship between SNPs and the translational regulation to its target genes is proposed. But, ascertaining biological function for each SNP often requires time-consuming, molecular biology experiments. Thus, analyzing the large number SNPs linked to any particular locus in practice requires a systematic bioinformatics evaluation and prioritization to narrow the set of likely functional candidate variants. Because most of the SNPs are in LD, the haplotype-based association studies are considered more powerful than the single SNP analysis to identify causal genetic variants underlying the etiology of complex diseases such as cancer [37], moreover, the use of tagSNPs that capture most of the haplotypic diversity in association studies has been suggested [38]. Though GWAS has yielded numerous SNPs or tagSNPs significantly associated with cancer, most of the tagSNPs are found in non-protein coding regions (intergenic and intron regions), identifying their functional and/or causal variants has an important limitation of GWAS data interpretation despite of assigning putative functionality to many other GWAS tagSNPs has only been successful when fine mapping around a known risk region was performed [39]–[41].

In the present study, we selected a putative functional tagSNP rs7560488 which can represent SNPs of the *DNMT3A1* promoter with high linkage disequilibrium, it is possible to identify genetic variation without genotyping every SNP in *DNMT3A1* promoter region and improve the efficiency of association. We observed that subjects carrying tagSNP rs7560488 TT genotypes exhibited significantly reduced gastric cancer risk compared with individuals with TC or CC genotype, indicating that allele T is a protective effect potentially exhibited by this tagSNP. Moreover, the assays we performed provided further evidence demonstrating that the TC and CC genotype associated with decreased expression levels of *DNMT3A1* mRNA in gastric cancer tissues, the results suggest that the *DNMT3A1* tagSNP rs7560488 T>C polymorphism may regulate the expression of *DNMT3A1* and thereby contribute to GC susceptibility. These data also indicated that DNMT3A1 may play a role in the progression of gastric cancer, but this finding needs to be confirmed by a larger population study. To our knowledge, this is the first report and demonstrates that the *DNMT3A1* transcription is directly influenced by functional tagSNP rs7560488 of *DNMT3A1* promoter region and the tagSNP rs7560488T>C was associated with a significantly increased risk of GC. Next, we performed an EMSA experiment to analyze the biological consequences of tagSNP rs7560488 polymorphism in BGC-823 cells. Both the T allele and the C allele probes showed two gel-shift bands, but competition experiments showed the binding affinity to the nuclear proteins by the T allele probe variant was greater than that seen with the C allele probe counterpart. So the enhanced DNA-protein binding ability of T allele may be responsible for the increased *DNMT3A1* promoter activity that we observed in our promoter assays. Super-EMSA experiment used AP-1 antibodies not got a super-gel shift indicated that transcription factors AP-1 may not involve in the formation of transcriptional complexes at the tagSNP rs7560488 site. Another novel result comes from the association between age and rs7560488 T>C polymorphism in stratified analysis implied that less than 60 years old were more susceptible to gastric cancer with tagSNP rs7560488 T>C. It is likely that *DNMT3A1* affects transcription of specific genes especially and changes in certain genes increase the risk of GC. Those results also show that the stronger risk factor for GC is tagSNP rs7560488 T>C, especially in young age.

Taken together, this study provides the first mechanistic insight into how this novel functional tagSNP rs7560488 T>C variant is significantly associated with risk of gastric cancer in a Chinese population. We found that the T to C change substantially altered transcriptional activity of *DNMT3A1* gene via influencing the binding of some transcriptional factors and so to change DNMT3A1 expression level, although a definite transcriptional factors remains to be established. Our findings provide an insight that *DNMT3A1* promoter rs7560488 T>C variation is a promising biomarker to evaluate the population susceptible to GC and provide valuable information toward future research in gastric cancer pathogenesis.
